# TERRA, hnRNP A1, and DNA-PKcs Interactions at Human Telomeres

**DOI:** 10.3389/fonc.2013.00091

**Published:** 2013-04-17

**Authors:** Phuong N. Le, David G. Maranon, Noelia H. Altina, Christine L. R. Battaglia, Susan M. Bailey

**Affiliations:** ^1^Department of Environmental and Radiological Health Sciences, Colorado State UniversityFort Collins, CO, USA

**Keywords:** TERRA, hnRNP A1, DNA-PKcs, hTR, telomeres, strand-specificity

## Abstract

Maintenance of telomeres, repetitive elements at eukaryotic chromosomal termini, and the end-capping structure and function they provide, are imperative for preserving genome integrity and stability. The discovery that telomeres are transcribed into telomere repeat containing RNA (TERRA) has revolutionized our view of this repetitive, rather unappreciated region of the genome. We have previously shown that the non-homologous end-joining, shelterin associated DNA dependent protein kinase catalytic subunit (DNA-PKcs) participates in mammalian telomeric end-capping, exclusively at telomeres created by leading-strand synthesis. Here, we explore potential roles of DNA-PKcs and its phosphorylation target heterogeneous nuclear ribonucleoprotein A1 (hnRNP A1) in the localization of TERRA at human telomeres. Evaluation of co-localized foci utilizing RNA-FISH and three-dimensional (3D) reconstruction strategies provided evidence that both inhibition of DNA-PKcs kinase activity and siRNA depletion of hnRNP A1 result in accumulation of TERRA at individual telomeres; depletion of hnRNP A1 also resulted in increased frequencies of fragile telomeres. These observations are consistent with previous demonstrations that decreased levels of the nonsense RNA-mediated decay factors SMG1 and UPF1 increase TERRA at telomeres and interfere with replication of leading-strand telomeres. We propose that hTR mediated stimulation of DNA-PKcs and subsequent phosphorylation of hnRNP A1 influences the cell cycle dependent distribution of TERRA at telomeres by contributing to the removal of TERRA from telomeres, an action important for progression of S-phase, and thereby facilitating efficient telomere replication and end-capping.

## Introduction

Telomeres are highly conserved tandem arrays of repetitive DNA sequence, TTAGGG in vertebrates (Meyne et al., 1989), that serve to protect the physical ends of linear chromosomes and prevent their detection as broken DNA, thereby evading an inappropriate damage response (de Lange, 2009). Adult human telomeres range in length from ∼5 to 15 kb and end with a 3′ single-stranded G-rich overhang of ∼12–300 nucleotides (Makarov et al., 1997; Wright et al., 1997; Zhao et al., 2008), a key feature for end-protection and extension. The telomeric single-stranded overhang can invade homologous double-stranded telomere tracks to create a telomere loop (t-loop) structure (Griffith et al., 1999). T-loop formation is facilitated by a complex of telomere-bound and associated proteins termed shelterin (de Lange, 2005; Palm and de Lange, 2008) that act together to sequester these natural DNA ends (reviewed in Martinez and Blasco, 2011). The G-rich single-stranded telomeric DNA is also subject to G-quadruplex formation (Parkinson et al., 2002), another structural solution that may contribute to end-protection. During development and later in some cell types including cancer and stem cells, the single-stranded G-rich overhang also provides the substrate required for elongation by telomerase, the specialized ribonucleoprotein (RNP) possessing reverse transcriptase activity (TERT) capable of catalyzing *de novo* telomere repeat addition utilizing an internal RNA template (TR) complementary to the telomeric DNA sequence (Greider and Blackburn, 1985, 1989).

As a consequence of residing at chromosomal termini, telomeres must also negotiate the “end-replication problem,” an issue resulting from the inherent inability of conventional DNA polymerases to replicate the extreme end of linear DNA (Olovnikov, 1971; Watson, 1972). In telomerase-negative human cells, the rate of telomere shortening is estimated to be ∼50–100 base pairs per cell division (Zhao et al., 2008). Telomeres synthesized by leading-strand semiconservative DNA replication are initially blunt-ended and so are not shortened compared to their parental DNA template. In contrast, newly replicated lagging-strand telomeres possess overhangs at the onset due to their requirement for an RNA primer, and therefore begin life shorter than their parental template, the degree of shortening being dependent on the position and removal of the terminal RNA primer (Chow et al., 2012). After processing to generate mature single-stranded 3′ overhangs, lagging-strand overhangs are ∼threefold longer than leading-strand overhangs (Zhao et al., 2008; Chow et al., 2012), the consequence being telomere shortening with each round of replication. Once telomeres become critically short, a state of irreversible cell cycle arrest known as cellular senescence is entered (Harley et al., 1990). The vast majority of cancer cells overcome this effective tumor suppressor barrier by re-activating telomerase (Kim et al., 1994), which adds ∼60 nucleotides to most chromosome ends (Zhao et al., 2009). Those tumors that do not express telomerase maintain telomere length via a recombination-based mechanism termed alternative lengthening of telomeres (ALT) (Murnane et al., 1994; Bryan et al., 1997; Dunham et al., 2000). In addition to tumor cells, telomerase has been shown to be active in germ line and stem cells, but is not present at sufficient levels in somatic cells to maintain telomere length (Kim et al., 1994; Mantell and Greider, 1994; Chiu et al., 1996; Hiyama et al., 1996; Wright et al., 1996).

Telomeres and their plethora of interacting partners must also create an environment refractory to DNA repair in order to maintain genomic stability (reviewed in O’Sullivan and Karlseder, 2010). Recent studies have demonstrated that telomeres and adjacent sub-telomeric regions (to ∼100 kb) are sensitive to double-strand breaks (DSBs) due to being deficient in their repair by non-homologous end-joining (NHEJ) (Kulkarni et al., 2010; Miller et al., 2011), and further that persistent DSBs near telomeres in normal cells, both *in vitro* and *in vivo*, contribute to aging and ionizing radiation-induced senescence (Fumagalli et al., 2012; Hewitt et al., 2012). The sensitivity of telomeric regions to unrepaired DSBs has also been proposed as an important contributor to chromosome instability in human cancer (Muraki et al., 2012). Nonetheless, the cell’s repair machinery has also been shown to be essential for telomere function. One such example is the NHEJ, shelterin associated DNA dependent protein kinase catalytic subunit (DNA-PKcs) that participates in mammalian telomeric end-capping, particularly at telomeres created by leading-strand synthesis (Bailey et al., 1999, 2001). Characterization of telomere dysfunction in DNA-PKcs deficient backgrounds provided evidence that DNA-PKcs kinase activity is critical (Bailey et al., 2004b), and further that autophosphorylation of *Prkdc* at the Threonine-2609 cluster (and not the Serine-2056 cluster) represents an important *in vivo* DNA-PKcs target at telomeres (Williams et al., 2009). Additionally, uncapped telomeres in such repair deficient backgrounds co-localized with γ-H2AX forming telomere dysfunction-induced foci (TIFs) (Takai et al., 2003), supporting their detection as DSBs and inappropriate triggering of a DNA damage response. It is also the case that many of the proposed mechanisms for generating 3′ single-stranded telomeric overhangs implicate DNA damage signaling and associated repair factors (Denchi and de Lange, 2007; Li et al., 2009). The SNMIB/Apollo 5′-to-3′ exonuclease that binds the shelterin component TRF2 (Freibaum and Counter, 2008), provides an interesting example in that it has been shown to be required for appropriate resection and formation of 3′ overhangs at leading-, but not lagging-strand telomeres, thereby protecting them from engaging the NHEJ pathway (Lam et al., 2010; Wu et al., 2012).

DNA-PKcs is a member of the phosphoinositide-3-kinase like kinase (PIKK) family that in humans also consists of ataxia telangiectasia-mutated (ATM), ATM/rad3-related (ATR), suppressor with morphogenetic effect on genitalia-1 (SMG1), mammalian target of rapamycin (mTOR), and transformation/transcription domain associated protein (TRRAP), many of which have been implicated in telomere function. For example, SMG factors have been shown to bind human telomeres and their depletion induces telomeric aberrations, including loss of telomeres and accumulation of telomeric RNA at telomeres (Azzalin et al., 2007). Importantly, SMG1 functions in the nonsense RNA-mediated decay (NMD) pathway via phosphorylation of the telomere associated eukaryotic helicase up-frameshift 1 (UPF1) (Yamashita et al., 2001; Azzalin et al., 2007; Isken and Maquat, 2008; Chawla et al., 2011). UPF1 is a downstream effector of SMG1 that can be activated by other PIKKs as well, including DNA-PKcs and ATR (Brumbaugh et al., 2004; Azzalin and Lingner, 2006; Muller et al., 2007), and is required for S-phase progression and genome stability (Azzalin and Lingner, 2006).

The majority of mammalian repetitive telomeric DNA is tightly packaged into nucleosomes (Pisano et al., 2008) and carries epigenetic marks characteristic of constitutive heterochromatin (reviewed in Schoeftner and Blasco, 2009). Telomeres therefore, have suffered from the misconception of being “junk” DNA and had certainly long been considered transcriptionally silent. However, telomeres from a variety of species including mammalian, are indeed transcribed into heterogeneous, non-coding transcripts, or telomere repeat containing RNA termed TERRA (Azzalin and Lingner, 2008; Schoeftner and Blasco, 2008). Chromatin bound TERRA specifically localizes at telomeres and exists predominantly as UUAGGG transcripts, thus are transcribed primarily from telomere C-rich/leading parental strands (Azzalin et al., 2007), that average around 200 bases in length (Porro et al., 2010). RNA-DNA hybrids of TERRA with telomeric DNA have been proposed (Luke et al., 2008). Telomeric G-rich RNA has been shown to form G-quartets (Randall and Griffith, 2009), and *in vitro* structural studies have demonstrated that telomeric RNA can specifically associate with telomeric DNA via formation of hybrid parallel G-quadruplex structures (Xu et al., 2008). Investigation of human TERRA RNA in living cells has provided the first *in vivo* evidence that TERRA RNA G-quadruplexes can localize to telomeres (Xu et al., 2010). A variety of functions have been proposed for TERRA, including regulation of telomerase activity, an appealing possibility as TERRA presumably duplexes with the complementary telomerase RNA template, hTR (Azzalin et al., 2007; Schoeftner and Blasco, 2008). However, the view that TERRA acts to inhibit telomerase has recently been challenged by the demonstration that telomere length is maintained independently of TERRA and highly transcribing telomeres (Farnung et al., 2012). TERRA, like other non-coding RNAs, may facilitate telomeric heterochromatin formation through its interactions with the shelterin component Telomere Repeat Factor 2 (TRF2), as well as with other proteins that facilitate heterochromatin formation, including the origin of recognition complex (ORC) and heterochromatin protein 1 (HP1) (Deng et al., 2009). TERRA interactions have also been proposed to promote telomere replication (Feuerhahn et al., 2010), particularly at leading-strand telomeres (Chawla et al., 2011), and facilitate end-capping function (Flynn et al., 2011).

Relatively little is known regarding the transcriptional regulation of TERRA, although it is it at least partially transcribed by RNA polymerase II (RNAPII) initiating at sub-telomeric CpG-rich promoters, and shelterin components appear to play key roles in regulating the process (Azzalin et al., 2007; Schoeftner and Blasco, 2008; Nergadze et al., 2009; Caslini, 2010). TERRA levels vary through the cell cycle, being lowest in late S-phase and peaking in early G1 (Porro et al., 2010), and TERRA can regulate its own transcription dependent on telomere length (Arnoult et al., 2012). TERRA repression in human cells is dependent on chromatin status of the telomeric region, as well as the nonsense mediated RNA decay (NMD) pathway, whose actions are restricted to the cytoplasm (Singh et al., 2007). Depletion of the NMD factors SMG1 and UPF1 resulted in the dramatic accumulation of telomere-bound TERRA, while total TERRA levels and turnover rate were not affected (Azzalin et al., 2007). Further, efficient replication of leading-strand telomeres has been shown to require human UPF1 (Chawla et al., 2011), as depletion of UPF1 resulted in fragile telomeres, a phenotype reflective of telomere replication associated defects (Sfeir et al., 2009), specifically involving leading-strand telomeres. Such studies provide additional support for strand-specific interactions at telomeres, as well as for telomere instability resulting from improper removal of telomere-bound TERRA. In human cells, poly(A) tails have been demonstrated on the fraction of TERRA transcripts *not* associated with chromatin (i.e., “free”), which contributed to their stability; TERRA transcripts associated with chromatin (i.e., “bound”) did not possess poly(A) tails, findings suggestive of distinct biological roles of free vs. bound TERRA (Porro et al., 2010).

The RNA binding protein heterogenous ribonuclear protein A1 (hnRNP A1) belongs to a large family of hnRNPs (A–U) (He and Smith, 2009) that function in shuttling mature RNA into the cytoplasm, and in mediating splice site selection during alternative splicing (Mayeda and Krainer, 1992; Martinez-Contreras et al., 2007). hnRNP A1 has also been shown to bind telomeric sequences, promote telomerase activity, and telomere length extension, as well as facilitate removal of replication protein A (RPA) from single-stranded telomeric DNA and participate in telomeric end-capping following replication (LaBranche et al., 1998; Ford et al., 2002; Zhang et al., 2006; Flynn et al., 2011). Other proposed functions of hnRNP A1 that may contribute to telomere replication include its ability to unwind G-quadruplexes (Zhang et al., 2006), and to interact with human telomerase (LaBranche et al., 1998) and telomerase RNA (Fiset and Chabot, 2001). Together with the fact that the consensus binding site of hnRNP A1 resembles TERRA (Burd and Dreyfuss, 1994), accumulating studies support an important role for hnRNP A1 in telomere RNA/TERRA function (de Silanes et al., 2010). Of particular interest to us in this regard were the demonstrations of direct hnRNP A1 phosphorylation by DNA-PKcs (Zhang et al., 2004), and of stimulation of DNA-PKcs dependent hnRNP A1 phosphorylation by hTR, the RNA template component of human telomerase; novel phosphorylation sites on hnRNP A1 targeted by DNA-PKcs were also identified (Ting et al., 2009).

Here, we interrogated potential roles of hTR stimulated DNA-PKcs dependent phosphorylation of hnRNP A1 in human telomeric end-capping structure and function involving TERRA. Evaluation of co-localized foci utilizing RNA-Fluorescence *In situ* Hybridization (FISH) and three-dimensional (3D) reconstruction strategies in conjunction with either inhibition of DNA-PKcs kinase activity or siRNA depletion of hnRNP A1 in human cells, revealed significant accumulation of TERRA at telomeres, which corresponded with an increased frequency of fragile telomeres (Sfeir et al., 2009) with reduced hnRNP A1. These results are consistent with those reported for depletion of SMG1 (Azzalin et al., 2007) and UPF1 (Chawla et al., 2011), and suggest that DNA-PKcs phosphorylation of hnRNP A1 influences the cell cycle dependent distribution of TERRA at telomeres. We propose that hTR/DNA-PKcs and hnRNP A1 interactions at telomeres contribute to the removal of chromatin bound TERRA, thereby facilitating efficient replication of telomeres and effective end-capping.

## Results

### DNA-PKcs dependent phosphorylation of hnRNP A1 is stimulated by hTR in human mammary epithelial cell lines

**Figure 1:**
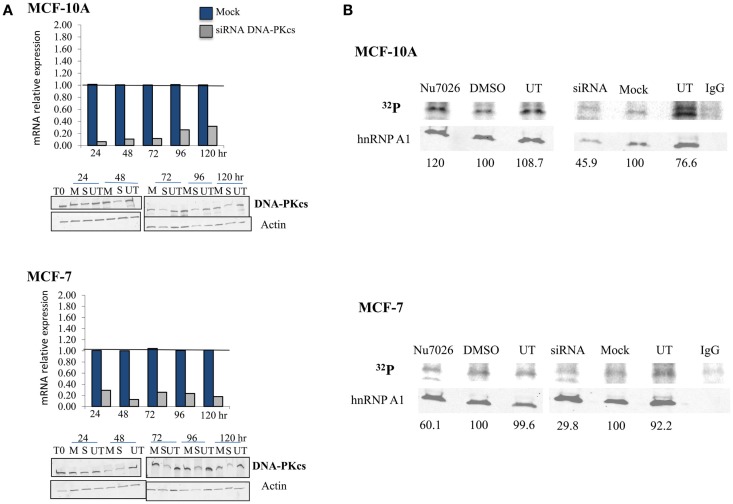
**DNA-PKcs dependent phosphorylation of hnRNP A1 in MCF-10A and MCF-7**. **(A)** DNA-PKcs siRNA knockdown. Real-time quantitative PCR assessment of DNA-PKcs mRNA relative expression from 24 to 120 h following transfection in mock (M), siRNA (S) treated cells, and the housekeeping gene Transferrin Receptor (TFRC). DNA-PKcs mRNA expression was normalized to TFRC levels in each sample and found to be maximally decreased at 24–48 h. DNA-PKcs protein levels were also assessed following siRNA transfection in mock (M), siRNA (S), or untreated (UT) cells over an identical time course; DNA-PKcs protein expression was normalized to the actin control. Optimal depletion of DNA-PKcs protein levels was observed at 72 h for both cell lines. An extended time course (to 240 h) monitored recovery of protein levels (not shown). **(B)** Overall phosphorylation status of hnRNP A1. ^32^P uptake experiments followed by immunoblotting demonstrated decreased hnRNP A1 ^32^P signal with DNA-PKcs depletion (siRNA) and kinase inhibition (NU7026) in MCF-7, and following DNA-PKcs siRNA silencing in MCF-10A; no decrease of hnRNP A1 ^32^P signal with DNA-PKcs kinase inhibition in MCF-10A was observed.

The hTR component of telomerase has previously been shown to stimulate DNA-PKcs dependent, site-specific phosphorylation of hnRNP A1 (Ting et al., 2009). To confirm DNA-PKcs dependent phosphorylation of hnRNP A1 in MCF-10A (“normal”) and MCF-7 (tumor) human mammary epithelial cell lines, we performed ^32^P uptake experiments to evaluate overall hnRNP A1 phosphorylation following DNA-PKcs depletion (siRNA) or kinase inhibition (NU7026). Consistent with our previous experience in other cell lines (Zhang et al., 2005), DNA-PKcs mRNA levels were reduced by 24–48 h and protein levels were maximally reduced at 72 h following siRNA transfection in both cell lines (Figure 1A). Relative protein expression of DNA-PKcs following siRNA silencing was comparable between MCF-10A and MCF-7 (1.66 ± 0.66 and 2.20 + 0.58 respectively), as determined from three independent protein isolations and immunoblots (data not shown).

Phosphorylated hnRNP A1, quantified per sample as the ratio of ^32^P signal to hnRNP A1 (i.e., in the same lane), decreased following DNA-PKcs siRNA knock down or kinase inhibition in MCF-7, indicating that hnRNP A1 is indeed an *in vivo* substrate for DNA-PKcs phosphorylation (Figure 1B). Similarly, decreased hnRNP A1 ^32^P signal was also observed in MCF-10A following siRNA depletion of DNA-PKcs, but no decrease in ^32^P signal following DNA-PKcs kinase inhibition was observed. This result likely reflects the hTR dependency of DNA-PKcs stimulation and subsequent phosphorylation of hnRNP A1, in that MCF-10A has significantly lower levels of telomerase and hTR than MCF-7 (Ramachandran et al., 2002; Ting et al., 2009); i.e., low levels of DNA-PKcs kinase stimulation by hTR in MCF-10A, therefore little effect of DNA-PKcs kinase inhibition on overall hnRNP A1 phosphorylation status. Additionally, because this assay reflects overall hnRNP A1 phosphorylation status, a reduction in low levels of DNA-PKcs dependent site-specific phosphorylation of hnRNP A1 may well be masked. Consistent with this view, decreased DNA-PKcs protein levels (siRNA) did result in reduced hnRNP A1 ^32^P signal, indicating that hnRNP A1 is a substrate for DNA-PKcs phosphorylation in MCF-10A, although it may not be particularly robust due to the low levels of hTR. It is also possible that other kinases targeting hnRNP A1 are affected by the loss of DNA-PKcs; e.g., ATM (Peng et al., 2005).

### TERRA co-localization at telomeres is influenced by hTR/DNA-PKcs and hnRNP A1 interactions

**Figure 2:**
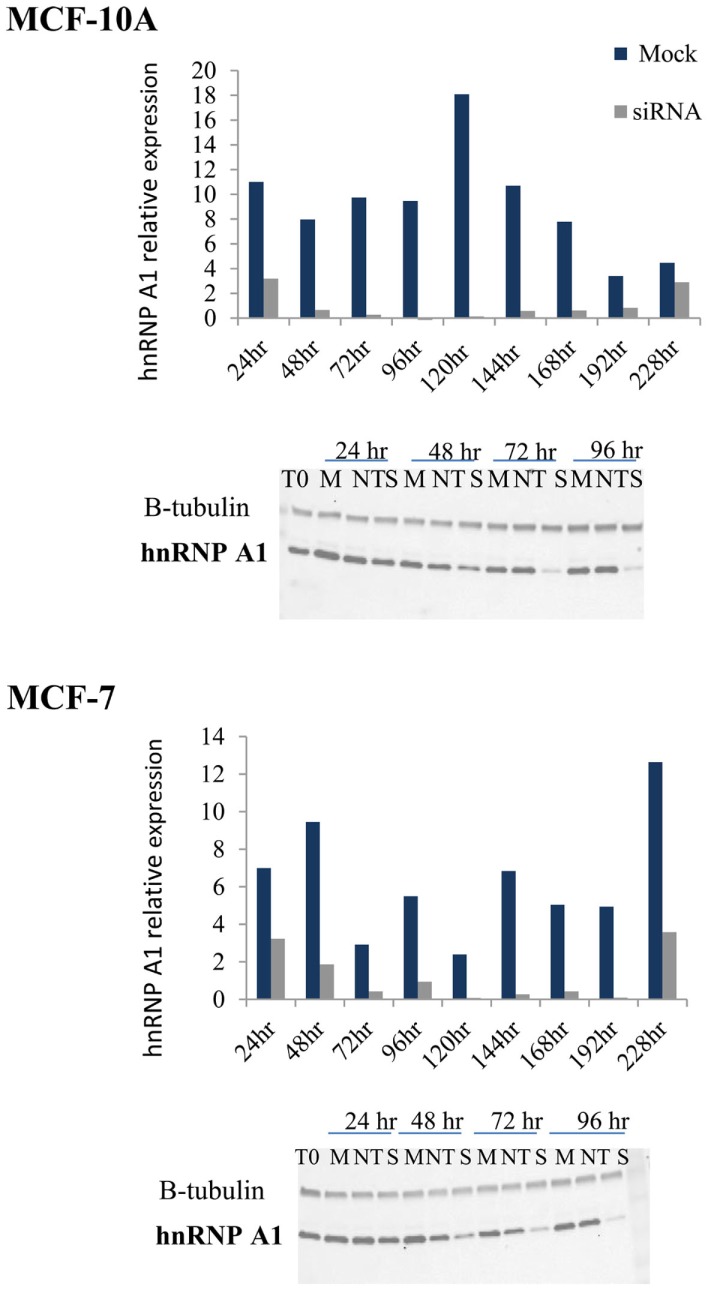
**hnRNP A1 siRNA knockdown in MCF-10A and MCF-7**. Following hnRNP A1 siRNA transfection, cells were harvested at various times (24–228 h) and hnRNP A1 protein levels evaluated (Western blot); graphs represent hnRNP A1 levels normalized to β-tubulin. Optimal knockdown of hnRNP A1 protein (∼90%) was achieved at 72 h for both cell lines (*n* = 4, each examined with two immunoblot analyses).

To evaluate the hTR/DNA-PKcs and hnRNP A1 dependency of TERRA co-localization to telomeres, we inhibited DNA-PKcs kinase activity (NU7026) or depleted hnRNP A1 protein levels (siRNA) in MCF-10A (low hTR) and MCF-7 (high hTR). Following hnRNP A1 siRNA transfection, cells were harvested at various times from 24 to 228 h and hnRNP A1 protein levels monitored relative to β-tubulin by immunoblotting (Figure 2). Optimal hnRNP A1 knockdown (∼90%) was achieved at 72 h for both cell lines. A non-target (NT) oligonucleotide sequence was also included to confirm minimal off-target effect of the siRNA knockdown; average 16.6% decrease (range 5.5–27%) in MCF-10A and a 3.3% decrease (range −3.6 to 6.7%) in MCF-7 was observed. Four independent collections were performed and immunoblot analysis was repeated twice per collection.

**Figure 3:**
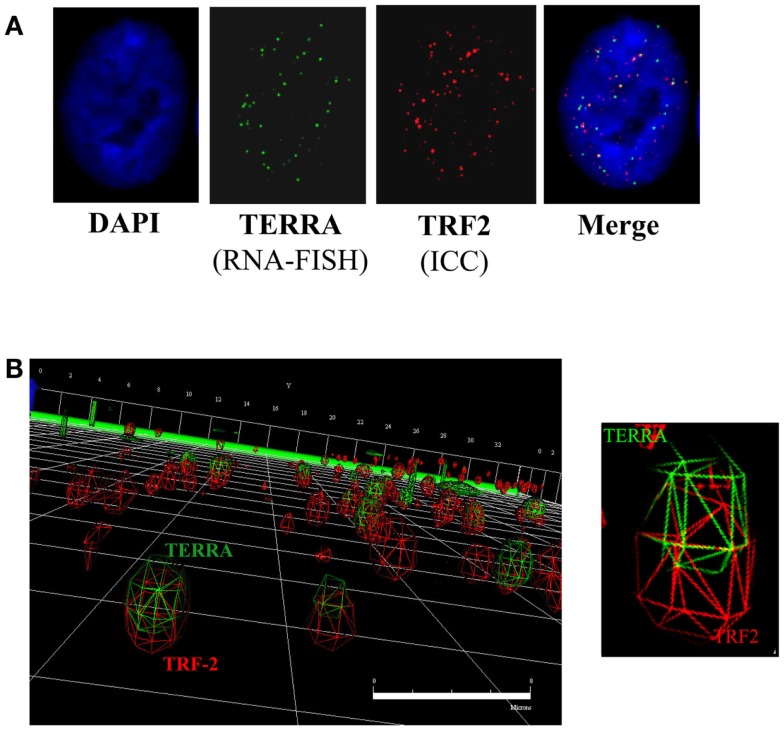
**TERRA co-localization at telomeres**. **(A)** 2D analysis of TERRA and TRF2 foci. Merging of the green (TERRA) and red (TRF2/telomeres) channels denotes potentially co-localized foci as yellow signals. **(B)** 3D analysis of TERRA and TRF2 foci. Deconvolution and 3D reconstruction of 22 stacks per cell nuclei provided a high-resolution perspective of TERRA co-localization at individual telomeres. Navigation of the 3D image provided a defined representation of telomere location throughout the cell nucleus, as well as TERRA distribution; i.e., either bound (co-localized with telomere) or free (not co-localized with telomere). Scale bar = 8 microns.

Three-dimensional (3D) reconstruction of TRF2 (telomeres; red) and TERRA (RNA-FISH; green) foci was performed on controls and following DNA-PKcs kinase inhibition (NU7026) or hnRNP A1 siRNA depletion (Figure 3). Co-localization of TRF2 and TERRA foci was microscopically evaluated based on merged yellow signals (2D), then confirmed by 3D deconvolution and reconstruction image analysis as a single focus sharing both red and green fluorochrome radii. Only cells presenting obvious TERRA foci, irrespective of intensity or number, were selected for analysis. Considering that TERRA levels are cell cycle dependent, being highest in G1 and diminishing into S-phase, and that the number of telomere (TRF2) signals were monitored, the majority of cells scored were presumed to be in G1 to early/mid S-phase, when TERRA associates with telomeres (Porro et al., 2010), and telomeres are widely distributed throughout the nucleus (Chuang et al., 2004). Pearson’s, Overlap and Mander’s coefficients were calculated to evaluate the degree of foci co-localization; values confirmed that the two molecules were in very close physical proximity to one another.

**Figure 4:**
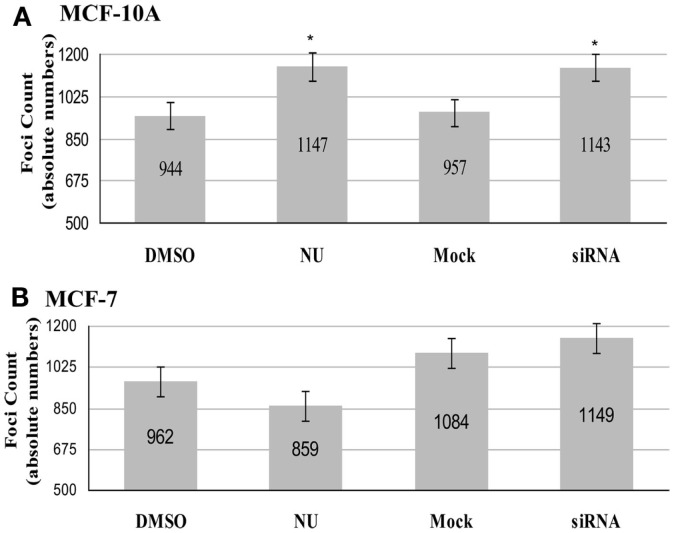
**Total number of co-localized TRF2/TERRA foci**. Experimental groups consisted of: control (DMSO) and DNA-PK inhibitor (NU7026); control (mock) and hnRNP A1 depleted (siRNA). **(A)** In MCF-10A, statistically significant increases in co-localization of TERRA at telomeres (TRF2) were observed for both NU7026 and siRNA treatments (*p < 0.05). **(B)** In MCF-7, no statistically significant differences in co-localization of TERRA at telomeres (TRF2) were observed for either NU7026 or siRNA treatment (*p* > 0.05). Data are ± SEM for *n* = 40 TERRA positive cells.

In MCF-10A (Figure 4A), comparison of co-localized foci in 40 TERRA positive cells within each treatment group revealed that both controls (DMSO and mock) had similar total numbers of co-localized foci (944 and 957; average number co-localized foci/cell 24), and both treatments (NU and siRNA) resulted in similar, significant increases compared to their respective controls (1147 and 1143; average number co-localized foci/cell 29). DNA-PKcs kinase inhibition resulted in 22% more co-localized foci in treated vs. DMSO controls, and hnRNP A1 siRNA depletion resulted in a 19% increase compared to the mock control; both increases were statistically significant (*p* = 0.002 and 0.007 respectively).

In MCF-7 (Figure 4B), the tumorigenic counterpart to MCF-10A, comparison of co-localized foci in 40 TERRA positive cells within each group revealed no significant effect of either treatment. The total number of co-localized foci decreased in DNA-PKcs kinase inhibited cells (859; average number of co-localized foci/cell 21) compared to DMSO control (962; average number of co-localized foci/cell 24), but was not statistically significant (*p* = 0.1352). Similarly, the total number of co-localized foci increased in the hnRNP A1 siRNA depleted cells (1149; average number co-localized foci/cell 29) compared to mock control (1084; average number co-localized foci/cell 27), but was not significant (*p* = 0.3429).

### Total TERRA foci are influenced by hTR/DNA-PKcs and hnRNP A1 interactions

**Figure 5:**
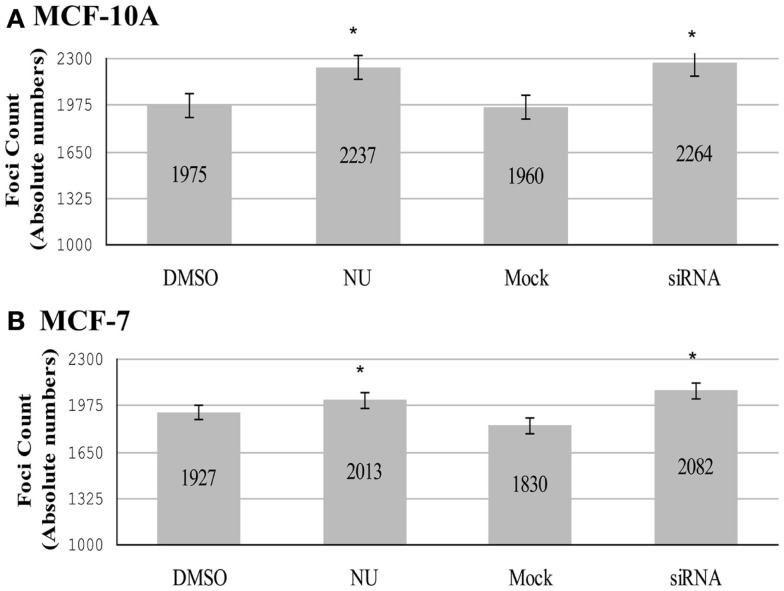
**Total number of TERRA foci (bound and free)**. Experimental groups consisted of: control (DMSO) and DNA-PK inhibitor (NU7026); control (mock) and hnRNP A1 depleted (siRNA). **(A)** In MCF-10A, statistically significant increases in the total number of TERRA foci were observed for both NU7026 and siRNA treatments (*p < 0.05). **(B)** Statistically significant increases in the total number of TERRA foci were also observed in MCF-7 for both treatments (*p < 0.05). Data are ± SEM for *n* = 40 TERRA positive cells.

Analysis of individual TERRA foci utilizing 3D reconstruction strategies revealed that DNA-PKcs kinase inhibition (NU7026) and depletion of hnRNP A1 (siRNA) significantly increased the total number of TERRA foci (co-localized/bound and free) compared to their respective DMSO or mock controls in both MCF-10A (Figure 5A) and MCF-7 cell lines (Figure 5B). Inhibition of DNA-PKcs kinase activity (NU) in MCF-10A resulted in a significant increase of TERRA foci over the DMSO control: total number of foci increased from 1975 to 2237; average foci per cell increased from 49 to 56 (*p* = 0.002). Similarly, depletion of hnRNP A1 (siRNA) in MCF-10A resulted in a significant increase of TERRA foci compared to the control (mock): total number of foci increased from 1960 to 2264; average foci per cell increased from 49 to 57 (*p* = 0.0004). In MCF-7, inhibition of DNA-PKcs kinase activity (NU) resulted in a significant increase of TERRA foci compared to the control (DMSO): total number of foci increased from 1927 to 2013; average foci per cell increased from 48 to 50 (*p* = 0.029). Depletion of hnRNP A1 (siRNA) in MCF-7 resulted in a significant increase of TERRA foci compared to the mock control: total number of foci increased from 1830 to 2082; average foci per cell increased from 46 to 52 (*p* = 0.031).

**Figure 6:**
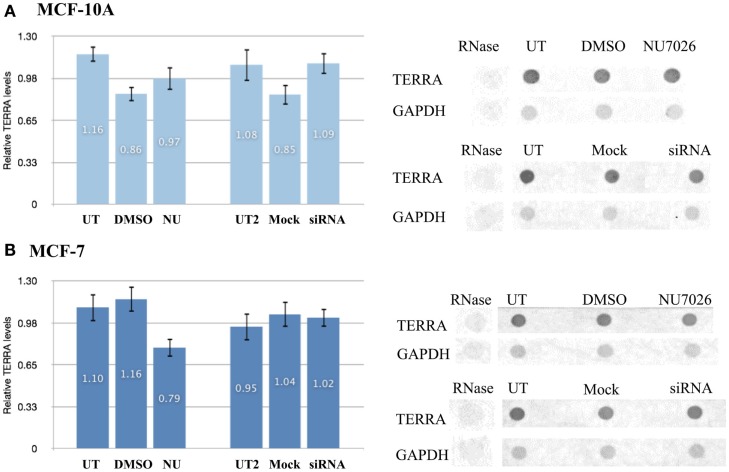
**Total TERRA levels**. Total RNA from unsynchronized cells was extracted and TERRA probed by dot blot and normalized to GAPDH (control). RNase treatment confirmed probe specificity for RNA. Comparisons examined for statistical analyses were: DNA-PKcs kinase inhibition (NU) vs. untreated (UT) and DMSO controls; or hnRNP A1 depletion (siRNA) vs. untreated (UT2) and mock controls. **(A)** Total levels of TERRA in MCF-10A were not significantly affected by either treatment (*p* > 0.05). **(B)** Total levels of TERRA in MCF-7 were not significantly affected by either treatment (*p* > 0.05). Data are ± SEM: for *n* = 3.

Comparison of the total number of TERRA foci (co-localized/bound and free) in MCF-10A and MCF-7 untreated controls (DMSO and mock) also revealed a statistically significant higher number of TERRA foci in MCF-10A (3935) than in MCF-7 (3757); average number of TERRA foci per cell being 49 and 46 respectively. In both treatment groups, MCF-7 also had fewer TERRA foci than MCF-10A (NU: 2013 vs. 2237; siRNA: 2082 vs. 2264). RNA dot blot analysis of total TERRA levels in *unsynchronized* MCF-10A and MCF-7 cells demonstrated that overall levels of TERRA were not significantly different between the two cell lines, and further that TERRA levels were not significantly affected by either DNA-PKcs kinase inhibition (NU) or depletion of hnRNP A1 (siRNA) compared to untreated and DMSO controls (Figure 6). The underlying reason for overall fewer total TERRA foci in MCF-7 compared to MCF-10A remains undetermined.

### Fragile telomeres are increased with depletion of hnRNP A1

**Figure 7:**
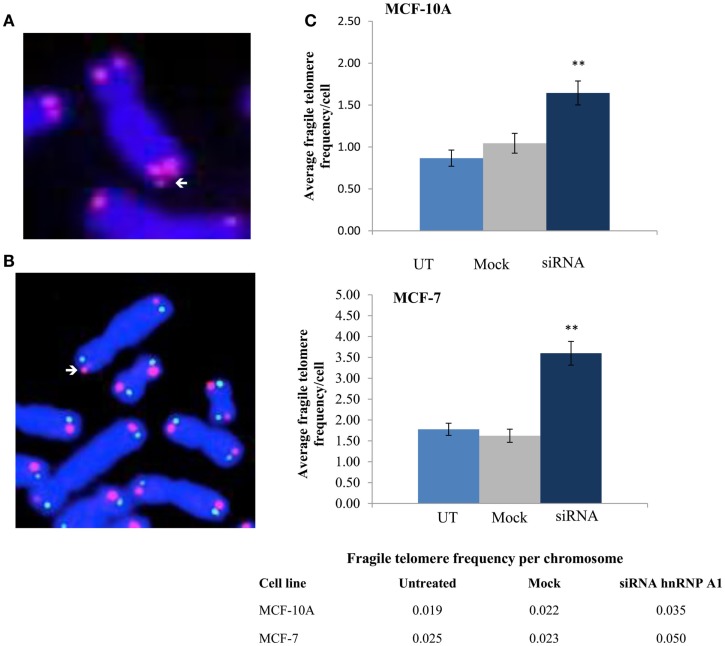
**Fragile telomere frequencies are increased with depletion of hnRNP A1**. **(A)** Standard telomere FISH analysis revealed significantly elevated frequencies of fragile telomeres (arrows). **(B)** Two-color telomere CO-FISH was employed to evaluate strand-specificity of fragile telomeres, however no significant preference for leading- vs. lagging-strand fragile telomeres was observed (*p* > 0.05; data not shown). **(C)** Induced fragile telomere frequencies per cell and per chromosome in both MCF-10A and MCF-7 were significantly elevated following hnRNP A1 depletion (siRNA) as compared to mock controls (**p < 0.01).

To investigate whether hTR/DNA-PKcs and hnRNP A1 interactions influenced efficient telomere replication and end-capping, we analyzed a variety of telomere dysfunction endpoints including fragile telomeres, as well as conventional chromosome aberrations following siRNA depletion of hnRNP A1. Standard telomere FISH analysis revealed significantly elevated frequencies of fragile telomeres, defined as extended (“stringy”) individual telomere signals, or as two distinct telomere signals on the same chromatid (doublets), in both MCF-10A and MCF-7 (Figure 7). Fragile telomere frequencies per cell were: MCF-10A UT 0.867, mock 1.044, siRNA 1.644, *p* < 0.01; and MCF-7 UT 1.778, mock 1.622, siRNA 3.60, *p* < 0.01. Two-color Chromosome-Orientation (CO)-FISH strategies (Bailey et al., 2004c, 2010) were also employed to evaluate potential strand-specificity for the incidence of fragile telomeres upon hnRNP A1 depletion, however no significant preference for leading- vs. lagging-strand was evident (*p* > 0,05; data not shown). Telomere signal free ends (SFEs) were also monitored, but no significant differences were seen (data not shown). Additionally, telomere sister chromatid exchange (T-SCE; Bailey et al., 2004a) frequencies were examined and no significant differences were observed (data not shown). There were also no significant increases in telomere fusion, or any chromosome or chromatid-type aberrations. Together, these results further support hTR/DNA-PKcs and hnRNP A1 participation in TERRA removal from telomeres facilitating efficient telomere replication and maintaining telomere stability.

## Discussion

Intrigued by the demonstrations of hnRNP A1 phosphorylation by DNA-PKcs, a post-translational modification shown to be stimulated by the RNA template component of human telomerase hTR (Zhang et al., 2004; Ting et al., 2009), we investigated potential roles of hTR/DNA-PKcs dependent phosphorylation of hnRNP A1 in human telomeric end-capping structure and function involving telomeric RNA (TERRA) distribution at telomeres. Interestingly, evaluation of co-localized foci utilizing 3D reconstruction strategies revealed a highly conserved orientation of telomeres/TRF2 and TERRA, an observation conceivably consistent with reports of non-random organization of mammalian telomeres (Chuang et al., 2004), telomere attachment to fixed subnuclear structures (de Lange, 1992), and/or constrained telomere movement (Wang et al., 2008).

Increased recombination at common fragile sites has been reported (Glover and Stein, 1987; Feichtinger and Schmid, 1989), and although telomeres do not exactly fit the classic definition of common chromosomal fragile sites (Le Beau, 1986; Sutherland and Richards, 1995), they do exhibit features of rare fragile sites in that they experience instability under replication stress. We found that both inhibition of DNA-PKcs kinase activity and siRNA depletion of hnRNP A1 resulted in significant accumulation of TERRA at individual telomeres, and further that depletion of hnRNP A1 increased frequencies of fragile telomeres. These observations are consistent with previous demonstrations that decreased levels of the nonsense RNA-mediated decay (NMD) factors SMG1 and UPF1 increased TERRA localization at telomeres (Azzalin et al., 2007) and interfered with replication of leading-strand telomeres (Chawla et al., 2011). However, no strand-specificity for fragile telomeres was observed upon loss of hnRNP A1, suggesting that hnRNP A1 operates at all telomeres to facilitate efficient telomere replication.

It is noteworthy that in both MCF-10A and MCF-7 under conditions of decreased DNA-PKcs and decreased hnRNP A1 phosphorylation, 30–60% of hnRNP A1 remained phosphorylated, most likely reflecting other protein kinases playing a role as previously proposed (Ting et al., 2009). Potential candidates for phosphorylating hnRNP A1 include protein kinase A, p38 MAP Kinase, protein kinase C, and casein kinase (Cobianchi et al., 1993; Municio et al., 1995; Gao et al., 2000; Shimada et al., 2009). Further, DNA-PKcs and ATM share many phosphorylation targets, including γ-H2AX (Wang et al., 2005) and Replication Protein A (RPA) (Brush et al., 1994, 1996; Boubnov and Weaver, 1995; Gately et al., 1998). Indeed, less phosphorylated hnRNP A1 remained following depletion of DNA-PKcs (siRNA; 30–45%) compared to DNA-PKcs kinase inhibition (NU; 60–100%), consistent with the observation that siRNA knockdown of DNA-PKcs also reduces ATM levels (Peng et al., 2005).

The dramatically different levels of hTR in MCF-10A vs. MCF-7 cell lines (Ramachandran et al., 2002) influenced observed DNA-PKcs mediated hnRNP A1 phosphorylation and TERRA co-localization at telomeres. We demonstrated that diminution of either DNA-PKcs kinase activity or hnRNP A1 levels in MCF-10A resulted in TERRA remaining at, or accumulating on telomeres, suggesting that hTR stimulation of DNA-PKcs phosphorylation of hnRNP A1 is sufficient in MCF-10A to help regulate TERRA distribution at telomeres. The observed lack of significant effect of inhibiting DNA-PKcs kinase activity or depleting hnRNP A1 on co-localization of TERRA at telomeres in MCF-7 is consistent with the considerably higher levels of hTR in MCF-7 as compared to MCF-10A, as hTR not only stimulates DNA-PKcs dependent phosphorylation of hnRNP A1 (Ting et al., 2009), hTR also associates with TERRA (Schoeftner and Blasco, 2008). The high levels of hTR in MCF-7 likely act to sequester TERRA, limiting its availability for co-localization to telomeres; thus depletion of hnRNP A1 and reduced ability to remove TERRA from telomeres had little effect. Such a scenario would also result in less available “free” hTR for stimulation of DNA-PKcs and subsequent phosphorylation of hnRNP A1; thus inhibiting DNA-PKcs kinase activity had little effect. Together, these results support hTR, DNA-PKcs, and hnRNP A1 levels and interactions influencing TERRA distribution on telomeres.

The observation of fewer total TERRA foci in MCF-7 than in MCF-10A was somewhat surprising considering that MCF-7 has considerably more chromosomes than MCF-10A (2*N* = 48 vs. 66–88), however decreased TERRA has been reported in cancer (Schoeftner and Blasco, 2008), suggestive of disrupted TERRA regulation. Therefore these results could reflect differences in TERRA degradation, and/or the faster cell cycle time for MCF-7 compared to MCF-10A and the variation of TERRA levels with phase of the cell cycle (Porro et al., 2010; Flynn et al., 2011). However, the majority of cells scored here were presumed to be in G1/early-to-mid S-phase, when TERRA levels are highest and TERRA foci most obvious; cells without obvious TERRA foci were not scored. When taken together with our observations of no change in co-localization of TERRA at telomeres (bound fraction) in MCF-7 with either treatment (Figure 4B), the increase of total TERRA foci in MCF-7 with both treatments reflects increases in the free (*not* co-localized/bound) fraction of TERRA. This finding is consistent with the high levels of hTR in MCF-7 acting to hinder TERRA association with telomeres.

**Figure 8:**
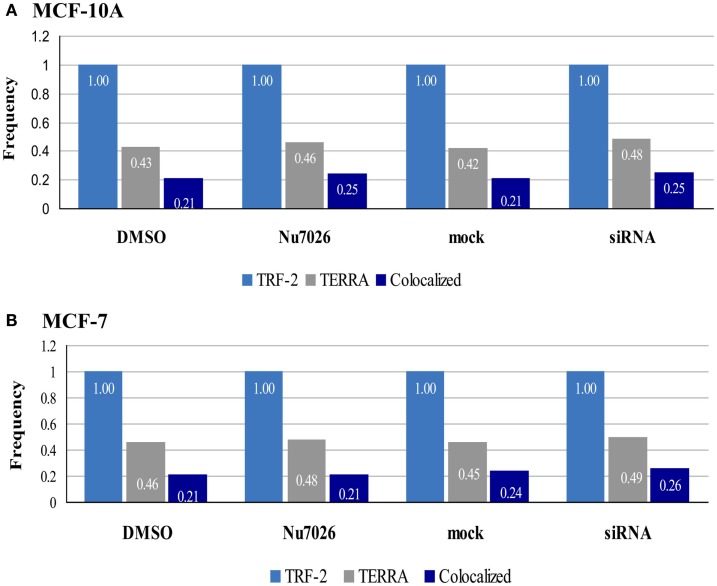
**Frequencies of TRF2, TERRA, and co-localized foci in MCF-10A and MCF-7**. 3D reconstruction and analysis of co-localization facilitated distinction between bound TERRA (TERRA foci co-localized at telomeres = dark blue bar), and free TERRA (difference between total TERRA foci = gray bar and co-localized/bound TERRA = dark blue bar). Number of telomere foci are normalized to 1.00 (light blue bar). Overall relationships are maintained, but the balance of free to bound TERRA is influenced by hTR, DNA-PKcs, and hnRNP A1 interactions.

It was also informative to consider the overall frequencies of TRF2 (telomeres), TERRA, and co-localized foci (Figure 8) as determined by our 3D reconstructions, as it became readily apparent that although DNA-PKcs kinase inhibition and depletion of hnRNP A1 resulted in variation, TERRA foci represented roughly half the number of telomeric foci. Moreover, of the TERRA that was present, about half was co-localized to telomeres (i.e., “bound”), indicating the other ∼half was “free” (i.e., not “bound” to telomeres). Additionally, ∼25% of the telomeres had TERRA co-localized, consistent with not all telomeres having TERRA associated with them. Therefore, our results suggest that the ratio or balance of free to telomere-bound TERRA is influenced by hTR, DNA-PKcs, and hnRNP A1 interactions.

**Figure 9:**
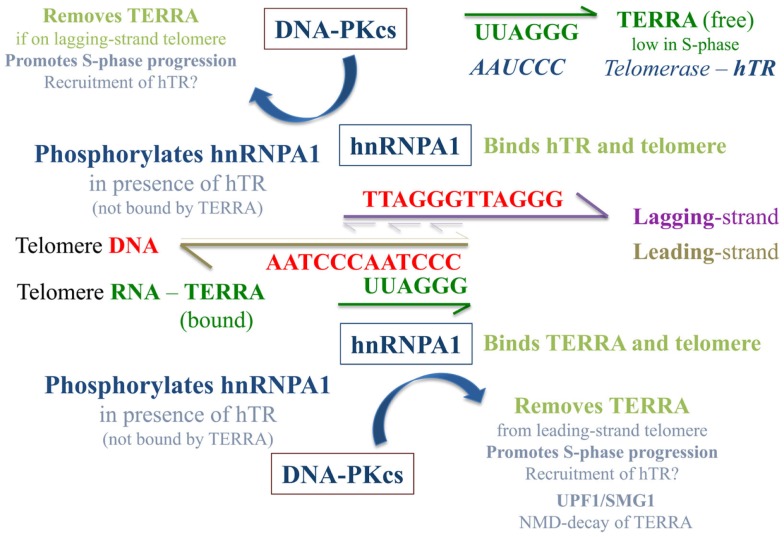
**Speculative model of TERRA, hnRNP A1, and hTR/DNA-PKcs interactions at telomeres**. The presence of “free” hTR (not associated with TERRA) stimulates DNA-PKcs phosphorylation of hnRNP A1, an event that contributes to the removal of TERRA from telomeres and may be especially important at leading-strand telomeres due to sequence complementary with TERRA. hnRNP A1 shuttling of TERRA off telomeres and out of the nucleus to the cytoplasm may aid the NMD pathway of TERRA degradation and serve to regulate TERRA levels in a cell cycle dependent manner. This supposition is supported by previous demonstrations of SMG1 stimulation and UPF1 action at leading-strand telomeres (Chawla et al., 2011). As TERRA is also complementary to hTR, when TERRA levels are low in S-phase, hnRNP A1 may also aid in the recruitment of hTR to its 3′ single-stranded overhang/substrate on newly replicated lagging-strand telomeres. When TERRA levels are high (e.g., in G1), sequestration of hTR by TERRA limits TERRA localization to telomeres, and influences the balance of free to telomere-bound TERRA.

Since hnRNP A1 associates with telomeric DNA and can interact with both telomeric RNA (TERRA) (de Silanes et al., 2010) and telomerase RNA (hTR) (Fiset and Chabot, 2001), we suggest a model in which hTR stimulated DNA-PKcs phosphorylation of hnRNP A1 serves to promote removal of “bound” TERRA from telomeres (Figure 9). It was recently proposed that TERRA shuttles hnRNP A1 off telomeres to facilitate a RPA-to-POT1 switch on single-stranded telomeric DNA after replication to facilitate end-capping (Flynn et al., 2011). We propose that hnRNP A1 may also shuttle TERRA off telomeres to facilitate efficient replication. Due to sequence complementarity, it may be especially important to remove TERRA from leading-strand telomeres and/or degrade TERRA sufficiently via UPF1 and the NMD pathway to promote S-phase progression. As TERRA is also complementary to hTR, it is also possible that hnRNP A1 participates in recruitment of free hTR to its 3′ single-stranded overhang/substrate on lagging-strand telomeres. Such interactions at telomeres, although requiring further examination, begin to address potential strand-specific differences between leading- vs. lagging-strand telomeres, which may serve to promote and/or complete their particular replication and/or extension, as well as their processing for generation of terminal single-stranded overhangs.

Lastly, we speculate that TERRA sequestration by hTR hinders TERRA localization to telomeres, such that the balance of free to telomere-bound TERRA is influenced by hTR. Likewise, the balance of free hTR to that associated with TERRA influences DNA-PKcs and hnRNP A1 interactions. In G1-phase for example, when TERRA levels are high, much of the hTR would be associated with TERRA. In S-phase, as TERRA levels diminish, more hTR would become available for stimulation of DNA-PKcs and site-specific hnRNP A1 phosphorylation, as well as for recruitment to telomeric single-stranded overhangs. We propose that DNA-PKcs and hnRNP A1 interactions are driven by hTR and TERRA associations, which ultimately serve to promote removal of telomere-bound TERRA, contributing to S-phase progression and efficient telomere replication.

Our contemporary view and appreciation of telomeres is far removed from the days of these deceptively simple terminal features being regarded as “junk” DNA. Although telomeres are strictly limited in sequence, restricted to location and constrained in movement, they provide chromosomal end-structure and function critical to maintaining genomic integrity and stability. That telomeres are transcribed is remarkable in and of itself, but TERRA also introduces a plethora of novel mechanisms not only for its own and telomere regulation, but for the regulation of the myriad of telomere interacting partners as well. Telomeric coordination of replication, transcription, and repression of repair is nothing short of extraordinary.

## Materials and Methods

### Cell culture

The human mammary epithelial non-tumorigenic cell line, MCF-10A, was purchased from ATCC and cultured in 1:1 Dulbecco’s Modified Eagle’s Medium (DMEM)/Ham’s F12 growth media (Hyclone) supplemented with 5% FBS, 20 ng/ml Epidermal Growth Factor (EGF; Sigma), 0.5 μg/ml hydrocortisone (Sigma), 0.1 μg/ml cholera toxin (Sigma), 10 μg/ml insulin (Sigma), 1% Glutamax (Life Technologies) and 1% penicillin/streptomycin (Hyclone). The MCF-7 cell line (gift from L. Chubb, CSU Animal Cancer Center), originally derived from a human breast adenocarcinoma, was grown in Minimum Essential Media/Earle’s Balanced Salt Solution (MEM/EBSS; Hyclone) media supplemented with 10% fetal bovine serum (FBS; Sigma). Cells were grown at 37°C in a humidified incubator with 5% CO_2_ and passaged 1–2 times per week.

### Transfections and treatments

For small interfering RNA (siRNA) knockdowns, reverse transfection was performed as recommended by the Lipofectamine RNAimax manufacturer (Life Technologies). Cells were seeded at ∼50% confluency and treated with lipofectamine alone (mock) or with lipofectamine and siRNA oligonucleotide (treated); an untreated (UT) control was also included. For targeting of DNA-PKcs, cells were incubated with custom designed siRNAs: sense sequence GAUCGCACCUUACUCUGUUdTdT; antisense sequence AACAGAGUAAGGUGCGAUCdTdT (Dharmacon), at a final concentration of 25 nM as previously described (Peng et al., 2002). For targeting of hnRNP A1, cells were incubated with the ON-TARGET plus SMARTpool siRNA (Dharmacon) at a final concentration of 10 nM. Oligo sequences included: CGGAAACCUUGGUGUAGUU; GGGAAUGAAGCUUGUGUAU; CAACUUCGGUCGUGGAGGA; and UAGAAUUCCUUCAGGGUGA. Cells were collected at various time points following transfection and knockdown efficiencies were evaluated by real-time Polymerase Chain Reaction (PCR) and immunoblot analysis. Optimal knockdown of DNA-PKcs and hnRNP A1 in both cell lines (∼90%) was achieved at 72 h, which was subsequently used for all experiments. Four experimental harvests were performed (*n* = 4) and each was examined with two immunoblot analyses.

For DNA-PKcs kinase activity inhibition, cells were treated with the specific DNA-PKcs kinase activity inhibitor 2-(Morpholin-4-yl)-benzo[h]chromen-4-one (NU7026; Sigma) solubilized in Dimethyl Sulfoxide (DMSO) at a final concentration of 10 μM (v/v 0.1% DMSO). A DMSO control was also included. Cells were incubated for 24 h at 37°C.

### Cell lysis, protein quantification, and immunoblotting

MCF-10A and MCF-7 cell pellets were washed in phosphate buffered saline (PBS) and incubated in lysis buffer for 10 min on ice with periodic mixing. Lysis buffer included 50 mM Tris-HCL, 150 mM NaCl, 2 mM ethylenediaminetetraacetic acid (EDTA), 2 mM ethylene glycol tetraacetic acid (EGTA), 25 mM sodium fluoride, 25 mM β-glycerophosphate, 0.2% Triton X-100, 0.3% NP-40, and 0.1 mM sodium orthovanadate in water. Protease inhibitors were added directly before use, including 0.1 mM phenylmethylsulfonyl fluoride (PMSF), 5 μg/ml leupeptin, and 5 μg/ml aprotinin. For phosphorylation experiments, cells were rinsed with PBS, and lysis buffer with Halt phosphatase inhibitors (Pierce) was added directly to the tissue culture flask for 10 min on ice. The Bradford Assay was used to quantify total protein amounts (Bio-Rad). For immunoblots, protein samples were separated via Sodium Dodecyl Sulfate Solution Polyacrylamide Gel Electrophoresis (SDS-PAGE) and transferred to a methanol activated polyvinylidene fluoride (PVDF) membrane. The membrane was blocked for 1 h with 4% powdered milk in Tris-buffered saline with 0.1% Tween (TBST) at room temperature, with rotation, and rinsed once in TBST before incubating with primary antibody in 1% milk in TBST for 1 h at room temperature or overnight at 4°C with shaking. Primary antibody dilutions included hnRNP A1 (Abcam ab5832 clone 9H10) at a 1:1000 concentration, β-tubulin (Abcam ab6046) at a 1:1000 concentration, DNA-PKcs (NeoMarkers MS-423-P) at a 1:40 concentration and Actin (Abcam Ab3280) at 1:1000. The membrane was subsequently washed 4 times in 1X TBST for 5 min each at room temperature with shaking, incubated with secondary antibody in Licor Blocking Buffer (Odyssey) for 2 h at room temperature with shaking and then washed in TBST washes as above. Alexa Fluor 680 (Invitrogen A21058) and goat anti-rabbit IgG 800 (Thermo Scientific 35571) secondary antibodies were added at a 1:10,000 and 1:40,000 dilution respectively. Lastly, the membrane was imaged on the Odyssey Imaging System (Licor). Relative protein expression was measured as a ratio of the intensity of the treatment bands to the housekeeping band using Odyssey imaging software and accounting for background.

### Phosphorus-32 (^32^P) uptake assay for hnRNP A1 phosphorylation status

MCF-10A and MCF-7 cells were rinsed once and incubated with phosphate-free media Dulbecco’s Minimum Essential Media (DMEM; Invitrogen) for 24 h to deplete adenosine triphosphate (ATP) pools. 400 μCi of ^32^P (orthophosphate; Perkin Elmer) per 2 ml of media was added and incubated at 37°C for 4 h to allow for labeling of ATP pools, as previously described (Ting et al., 2009). Cells were subsequently lysed and total protein amount quantified via Bradford assay, followed by immunoprecipitation of hnRNP A1 and Sodium Dodecyl Sulfate Solution Polyacrylamide Gel Electrophoresis (SDS-PAGE). Phosphorylation of hnRNP A1 was quantified per sample as the ratio of ^32^P signal to hnRNP A1 (i.e., in the same lane).

### Immunoprecipitation of hnRNP A1 and SDS-PAGE

Immunoprecipitations were performed using the Direct IP kit (Pierce) as recommended by the manufacturer. Whole cell lysates were pre-cleared by incubating with control resin for 30 min with rocking. Approximately, 1000 μg of protein was incubated with 50 μg of antibody against hnRNP A1 (Abcam ab5832 clone 9H10) or IgG control (R&D Systems MAB004) for 1 h with rotation at room temperature. Samples were acetone precipitated, subsequently examined by SDS-PAGE and transferred to a PVDF membrane using a semi-dry transfer method performed at 15 V for 1.25 h. Membranes were exposed to a phosphor imaging screen (Kodak) for 48 h, which was imaged on a Storm 860 (GE Healthcare Life Sciences) and quantified using Image Quant (GE Healthcare Life Sciences) software. The same membrane was rewet and probed for total amounts of hnRNP A1 via immunoblotting (see Cell Lysis, Protein Quantification, and Immunoblotting).

### Quantitative real-time polymerase chain reaction

Total RNA was harvested from untreated, mock treated, and siRNA treated samples using the Qiagen RNeasy kit (Qiagen). RNA was quantified using a Nanodrop spectrophotometer and reverse transcribed using the Verso cDNA kit (Thermo Scientific). Real-time PCR was performed using SYBR green (Thermo Scientific) according to manufacturer’s protocol and performed using a Bio-Rad iCycler IQ. The real-time cycle was as follows: Cycle 1 at 95°C for 15 min, cycle 2 (40×) step 1 at 95°C for 15 s, step 2 at 59°C for 30 s, and step 3 at 72°C for 30 s. A melt curve was included to assess primer dimers and non-specific amplification as follows: cycle 3 at 95°C for 30 s, cycle 4 at 55°C for 30 s, and cycle 5 (80×) at 55°C for 10 s. Primers for amplification of human DNA-PKcs (Sigma) were added at a final concentration of 300 nM: forward sequence AGCAATGCACCGTTGTGGT; reverse sequence TCCTTCTTCAGGAGCTTCCA. Primers for amplification of transferrin receptor (TFRC) (Sigma) at a final concentration of 300 nM were included as a housekeeping gene: forward sequence CGCTGGTCAGTTCGTGATTA; reverse sequence GCATTCCCGAAATCTGTTGT. Relative DNA-PKcs mRNA expression was analyzed using the 2^− ΔΔCT^ method.

### RNA-fluorescence *in situ* hybridization and immunocytochemistry

Cells were grown on chamber slides, then washed with cytoskeleton (CSK) buffer for 30 s, fixed in 3% paraformaldehyde for 10 min and permeabilized with 0.5% Triton X-100 in CSK/vanadyl for 5 min. Following permeabilization, slides were blocked with 3% bovine serum albumin (BSA) for 1 h, then incubated for 1 h with mouse anti-telomere repeat factor 2 (TRF2) primary antibody (Imgenex; San Diego, CA, USA) followed by incubation with anti-mouse AlexaFluor-594 secondary antibody (Life Technologies) for 45 min. Slides were washed once with 1× PBS, incubated overnight with a FITC labeled peptide nucleic acid (PNA) (Bio-Synthesis Inc, Lewisville, TX, USA) telomere probe (CCCTAA)_7_ complementary to TERRA and sequentially washed in 50% formamide/2× saline sodium citrate (SSC), 2× SSC, and 2× SSC/NP-40 at 39^°^C for 2.3 min each. Lastly, the slides were mounted with ProLong Gold Antifade with 4′,6-diamidino-2-phenylindole dihydrochloride (DAPI; Life Technologies).

### Microscopy and imaging

Images were acquired and analyzed using a Zeiss Axio Imager.Z2 epi-fluorescent microscope running Metamorph software (Molecular Devices). For each cell, 22-stacked images taken at 0.2 μm intervals, per channel, were obtained with a 1.25 numerical aperture 100× oil immersion objective then analyzed by 3D deconvolution and reconstruction. ImageJ software (http://rsb.info.nih.gov./ij/) and JACoP plugin (National Institute of Health; NIH) were used to evaluate co-localization of TRF2 (telomeres) and TERRA foci from 3D images in the red channel for TRF2 (anti-mouse AlexaFluor-594; Life Technologies) and the green channel for TERRA (FITC labeled PNA probe; Bio-Synthesis Inc, Lewisville, TX, USA). Several co-localization coefficients such as Pearson’s, Mander’s, and Overlap were calculated using the ImageJ and JACoP plugin, to assess the degree of signal co-localization in the 3D images and generate co-localization profiles for each individual cell. Additionally, ImageJ software was utilized for co-localization foci counts and evaluation of the foci numbers. Costes randomization was used to exclude any co-localization of pixels that might have occurred due to chance (Bolte and Cordelieres, 2006). For each condition, 40 TERRA positive cells were imaged (*n* = 40).

### RNA dot blot

RNA dot blots were performed as previously described with some modifications (Kafatos et al., 1979). Total RNA was extracted using a Qiagen RNeasy kit (Qiagen), and RNA quality was assessed by gel electrophoresis. GeneScreen Plus Nylon (Perkin Elmer) and Bio-Dot SF filter paper (Bio-Rad) were pre-wet in 20× SSC and assembled into a Bio-Rad Bio-Dot apparatus attached to a vacuum source. The membrane was rinsed twice in 10× SSC. About 7.5 μg of RNA was suspended in RNA denaturing solution [66% formamide, 21% Formaldehyde (37%), 13% 10× MOPs pH 7] and denatured for 5 min at 75°C. Equal volumes of 20× SSC were added. Samples were subsequently applied to the Bio-Dot apparatus and washed twice with 10× SSC. Vacuum was applied to dry the membrane. The membrane was then removed and placed in a UV Stratalinker 2400 (Stratagene) equipped with 254 nm bulbs for 25–50 s. The membrane was stained in 0.02% Methylene blue, 0.5 M Sodium Acetate, pH 5.2 in order to visualize and mark RNA dots. The membrane was then incubated in hybridization solution [2 mM vanadyl, 50% formamide, 30% 20× SSC, 1% 50× Denhardt’s solution, 0.25% SDS (20%), 0.1% or 250 μg/ml salmon sperm DNA, brought up to volume with ddH_2_0] for 2 h at 42°C. Next, the membrane was incubated with fresh hybridization buffer containing denatured PNA FITC labeled TERRA probe (CCCTAA)_7_ and FITC labeled-GAPDH PNA probe (DAKO) overnight at 42°C. Following incubation, the membrane was washed with shaking: (1) four times in 2× SSC at room temperature for 10 min each; (2) two times in 0.1× SSC 2, 0.1% SDS at 50°C for 30 min each; and (3) two times in 0.1× SSC, 0.5% SDS at 68°C for 30 min each. Membrane imaging was performed on a Storm 860 (GE Healthcare) and analysis was done using ImageJ software. Three experimental harvests were dot blotted and analyzed (*n* = 3).

### Fluorescence *in situ* hybridization and two-color chromosome-orientation FISH

Metaphase spreads were collected using standard cytogenetic techniques (Henegariu et al., 2001). Briefly, cells were incubated with 0.1 μg/ml colcemid, harvested and lysed with 75 mM KCL, fixed in 3:1 methanol: acetic acid and added to pre-cleaned slides. Slides were fixed in 3% paraformaldehyde, dehydrated through an ethanol series (75, 85, and 100%) and denatured in 70% formamide/2× saline sodium citrate (SSC) at 70°C for 2 min. For standard FISH, a G-rich telomere Peptide Nucleic Acid (PNA) probe (TTAGGG)_7_ labeled with Cy-3 (Bio-Synthesis Inc; Lewisville, TX, USA) was hybridized onto the slides at 37°C for 30–60 min. For two color CO-FISH, the C-rich telomere probe (CCCTAA)_7_ labeled with FITC was also hybridized onto the slide at 37°C overnight. Slides were then washed in 50% formamide/2×SSC, 2× SSC, and 0.1% NP-40/2× SSC at 43°C for 2.5 min each. Lastly, slides were mounted in Prolong Gold Antifade (Life Technologies) with DAPI.

CO-FISH methodology has been described previously (Bailey et al., 2010) and was used here with modification. Cells were incubated for a single round of replication in 5-bromo-2′-deoxyuridine and 5-bromo-2′-deoxy-cytosine (BrdU/BrdC) at a 3:1 ratio for a final concentration of 5 × 10^−5^ M. Prior to denaturing, slides were stained with Hoechst 33258 (0.5 μg/ml), exposed to Ultraviolet (UV) light in a Stratalinker outfitted with 365 nm bulbs for 35 min, then incubated with Exonuclease III (∼10 U) for 20 min at 25°C. Lastly, slides were hybridized with telomere probes as described above. For FISH and CO-FISH analyses, three independent experiments (*n* = 3) were performed and 30 metaphases were scored per experiment. The individual experiments were averaged for statistical analysis.

### Chromosome and telomere aberration scoring criteria

Chromosome aberrations were scored as classically defined. Clonal chromosome-type aberrations were not included in the final analysis, as our intent was to evaluate induced aberration frequencies. Chromatid-type gaps were defined as discontinuities in DAPI staining less than the width of a chromatid arm. Chromatid-type breaks were defined as a discontinuity in DAPI equal to or greater than the width of a chromatid. Telomere function was evaluated using several endpoints. Fragile telomeres were defined as one extended telomere signal (i.e., “stringy”) and/or duplicated telomere signals on the same sister chromatid within one signal width of one another. Telomere signals that did not meet these criteria were scored as interstitial telomere signals (ITS) and were not included as a “fragile” telomere. Signal free ends (SFE) were assessed following both FISH and CO-FISH using two different scoring criteria. First, SFEs were scored as a complete lack of telomere signal on one chromatid arm. Secondly, SFEs were scored as a single event only if the signal was missing from both sister chromatid arms, as would be expected with telomere shortening and subsequent replication. T-SCE were scored as a CO-FISH telomere signal split between the two sister chromatids. Telomere fusion was defined as two telomere signals merged into one at chromosome or chromatid rearrangement breakpoints.

### Statistical analysis

Experimental output for siRNA knockdowns were statistically analyzed using student *t*-test via GraphPad Prism statistical software (v5.0, La Jolla, CA, USA). All other statistical analyses were performed using SAS (SAS Institute Inc., Cary, NC, USA) and the Simple Differences Least Squares Means model to determine statistically significant trends in the data. All data is represented as the mean ± SEM. A *p*-value <0.05 was considered to be statistically significant.

## Conflict of Interest Statement

The authors declare that the research was conducted in the absence of any commercial or financial relationships that could be construed as a potential conflict of interest.
